# Fucosyltransferase 2: A Genetic Risk Factor for Intestinal Diseases

**DOI:** 10.3389/fmicb.2022.940196

**Published:** 2022-07-18

**Authors:** Mingyang Hu, Xiyun Zhang, Jinze Li, Luotong Chen, Xiaolin He, Tingting Sui

**Affiliations:** Key Laboratory of Zoonosis Research, Ministry of Education, Institute of Zoonosis, Jilin University, Changchun, China

**Keywords:** FUT2, histoblood group antigens, inflammatory bowel disease, intestinal microbiota, ulcerative colitis (UC), Crohn's disease

## Abstract

The fucosyltransferase 2 gene (*FUT2*) mediates the synthesis of histoblood group antigens (HBGA) that occur *in vivo* from multiple organs, particularly on the surface of intestinal epithelial cells and body fluids. To date, many studies have demonstrated that the interaction of HBGA with the host microbiota is the cause of pathogenesis of intestinal diseases, making *FUT2* non-secretor a risk factor for inflammatory bowel disease (IBD) due to the lack of HBGA. As HBGA also acts as an attachment site for norovirus (NoV) and rotavirus (RV), the non-secretor becomes a protective factor for both viral infections. In addition, the interaction of norovirus and rotavirus with symbiotic bacteria has been found to play an important role in regulating enteroviral infection in IBD. Given the current incomplete understanding of the complex phenomenon and the underlying pathogenesis of intestinal diseases such as IBD, it has recently been hypothesized that the *FUT2* gene regulates intestinal bacteria through attachment sites, may help to unravel the role of *FUT2* and intestinal flora in the mechanism of intestinal diseases in the future, and provide new ideas for the prevention and treatment of intestinal diseases through more in-depth studies.

## Introduction

ABO, known as the ABO blood group system, is found for classifying blood by specific antigens (agglutinogens) A and B on the surface of red blood cells. In the ABO blood group system, the antigen that eventually forms H antigens is a glycan chain catalyzed by a gene-encoded enzyme called fucosyltransferase (Barbé et al., 2018). In 1960, Watkins found that the antigen ABO is a carbohydrate, namely HBGA. The fucosyltransferase 2 (*FUT2*) gene mediates the synthesis of HBGA (Rouquier et al., 1995). In recent years, genome-wide association studies (GWAS) have highlighted the importance of *FUT2* biology, and HBGA was found to serve as an attachment site for both intestinal flora and norovirus (NoV) and rotavirus (RV), identifying different polymorphisms of this gene resulting in distinct secretor statuses associated with the development of pathophysiological conditions between intestinal diseases (Maroni et al., 2015; Iliev and Cadwell, 2021; Tarris et al., 2021). This leads to new hypotheses for the study of complex mechanisms of intestinal diseases. In this review, we aimed to provide a brief overview of the effect of *FUT2* on intestinal diseases, link HBGA to intestinal flora and enterovirus through some hypotheses about the way HBGA mediates intestinal flora, and investigate whether the *FUT2* gene can be used to infer susceptibility to intestinal diseases so that the prevention can be better personalized for individuals in different regions. Therefore, we focused on the function of different FUT2 genotypes in terms of intestinal flora. As an outlook, the association of *FUT2* with COVID-19 and psychiatric disorders is briefly discussed.

## The Biosynthesis of HBGA

### The Synthesis Process of HBGA

**Figure 1:**
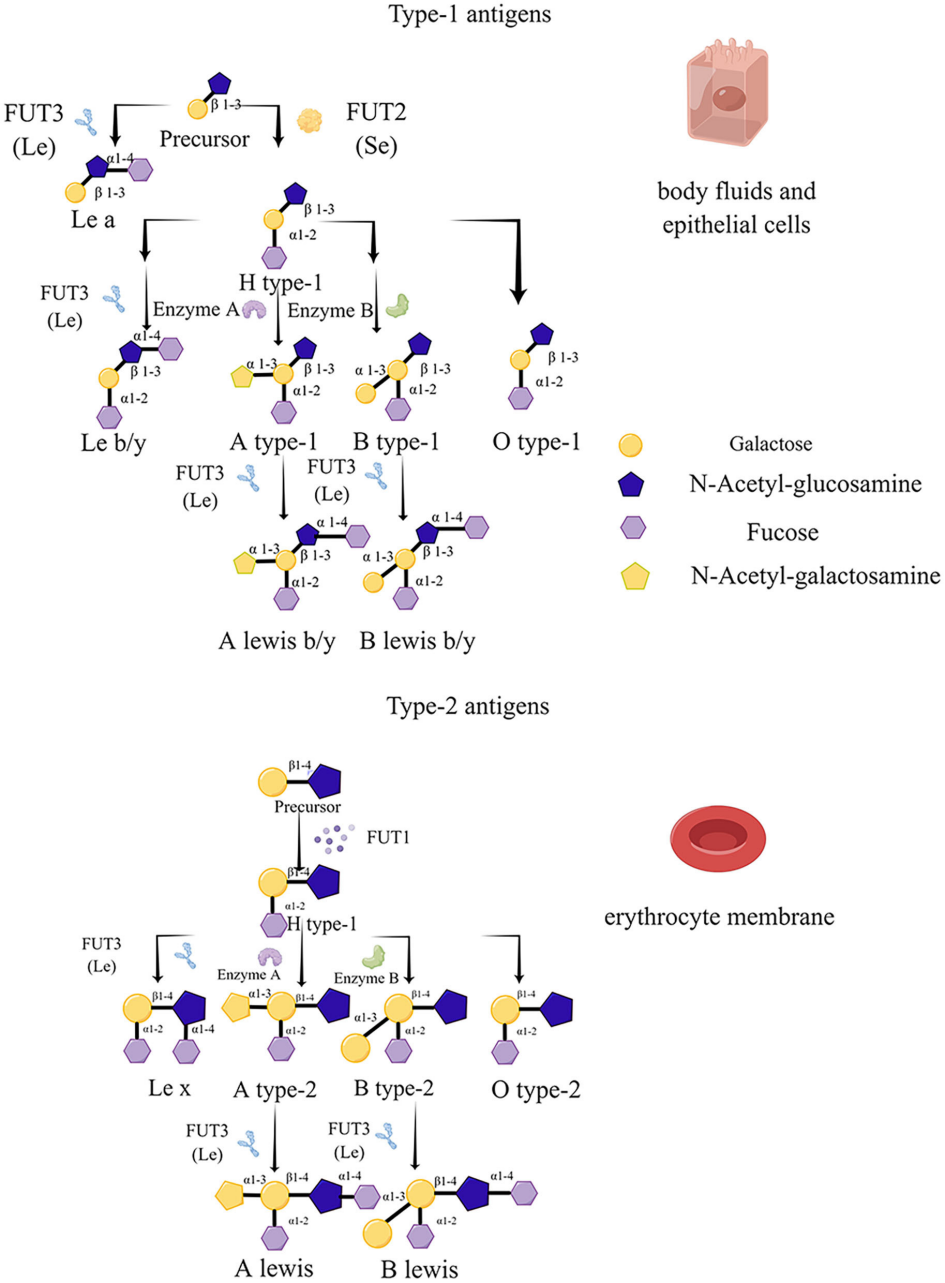
Biosynthesis routes and schematic structure of histo-blood group antigens (HBGAs). The antigen precursors on erythrocytes are mainly type 2 (N-Acetyl-galactosamine, N-Acetyl-Lac) catalyzed by the lactosyltransferases *FUT1* and *FUT3* for elongation, while those in other tissues and body fluids are type 1 precursors (Lacto-N-Biose, LNB), catalyzed by lactosyltransferase-2 (*FUT2*) and *FUT3* enzymes further catalyze the synthesis to produce H and Lewis antigens, as well as A and B blood groups by A and B enzymes.

In recent years, four types of fucosyltransferases have been distinguished according to the specific site of fucose addition to substrates, including α-1,2-, α-1,3/4-, α-1,6-, and O-fucosyltransferases. Among these types of fucosyltransferases*, FUT1, FUT2*, and *FUT3* catalyze α-1,2 and α-1,3/4 sites, respectively, which are indispensable for HBGA biosynthesis, and *FUT1* and *FUT2* play crucial catalytic roles in both types of H antigen formation (Kelly et al., 1995). Moreover, *FUT1* and *FUT2* have an apparent difference in acceptor specificity, and two types of H antigen precursors (type 1 and type 2) were regulated by the H enzyme encoded by the *FUT2* and *FUT1* gene, respectively, i.e., the acceptor specificity of *FUT1* is type 2 chains (galactose-β-1→ 4-*N*-acetyl-glucosamine, *N*-acetyl-lactosamine) (GBD 2017 Inflammatory Bowel Disease Collaborators, 2020); in contrast, *FUT2* gene-specified enzyme is more vigorous on type 1 (galactose-β-1→ 3-*N*-acetyl-glucosamine, lacto-*N*-biose) structures (Kyprianou et al., 1990). Two kinds of precursors serve as substrates for the *FUT2* and *FUT1* enzymes, which modify them by attaching an L-fucose to the galactose moiety *via* an α-1→ 2 linkage, generating type-1 and type-2 H antigens, respectively (Peña-Gil et al., 2021). However, if the *FUT3* enzyme modifies the precursor, Le^a^ (type 1 precursor) and Le^x^ (type 2 precursor) antigens are produced. Different from the formation of H-type antigens, the modification of *FUT3* involves the attachment of an L-fucose to N-acetyl-glucosamine with an α-1→ 4 linkage in the case of the type-1 precursor or an α-1→ 3 linkage in the case of the type-2 precursor (Monedero et al., 2018). Furthermore, H type-1 and type-2 antigens will be transformed to A and/or B blood groups catalyzed by two glycosyltransferases (A and B enzymes), which are coded by the ABO family. As a result, the *FUT3* enzyme can apply to the H type-1 and type-2 antigens generating Le^b^ and Le^y^ antigens (Marionneau et al., 2001; Tan and Jiang, 2014) (Figure 1).

### The Relevance of the HBGA and Secretor Status

In fact, HBGA can emerge from multiple organs *in vivo* probably. The reason is that the main function of the *FUT1* gene (H) is to control the form of antigen type 2 on the red blood cell; nevertheless, the *FUT2* gene (Se) mainly catalyzes the type 1 antigens of other mucosal tissues, body fluids, such as in saliva, breast milk and digestive juices (Ye and Yu, 2021), and epithelial cells. Therefore, we can determine, theoretically, when both *FUT1* and *FUT2* genes have normal expression functions, namely, the secretors genotype. Notably, it has been known that one-fifth of the population cannot express A, B, or H antigens in body fluids who are called as “non-secretor” (se). Actually, the existence of “non-secretor” is due to the nonfunctional mutations leading to the *FUT2* homozygous gene, which results in the improper synthesis of antigens. Thus, ABO antigens are undetectable in their epithelial tissues and body secretions. However, the standard quantity levels of ABO antigens exist on the membranes of erythrocytes with the *FUT1* gene being normal. Moreover, this phenomenon is consistent with the description of the tissue distribution of *FUT1* and *FUT2*. In contrast, if the *FUT1* gene is a recessive homozygote, a special o-type named Bombay type may occur.

Remarkably, the secretor status of the *FUT2* gene was suggested as a close correlation with a variety of gastrointestinal diseases, including IBD (Tang et al., 2021), and diseases caused by viruses, mainly norovirus (Nov) (Lindesmith et al., 2003; Currier et al., 2015) and rotavirus (RV) (Galeev et al., 2021). In fact, the reason behind this phenotype determines the presence or absence of HBGA, which consists of an attachment site and a carbon source of intestinal microbiota (Tong et al., 2014; Kononova, 2017), and the major binding saccharide of some viruses, including NoV (Chen et al., 2011) and RV (Sharma et al., 2020). Recently, alteration of the composition and function of the gut microbiota, namely dysbiosis, which was suggested, may greatly contribute to the pathogenesis of IBD. Moreover, the interaction between enteric virus and commensal bacteria seem to play a significant role in the modulation of enteric virus infections in IBD. Due to the association with HBGA and enteric virus, GWAS has demonstrated the association between IBD and single nucleotide polymorphism (SNPs) of the *FUT2* and *FUT3* genes, which drive the synthesis of HBGA, and ligands for NoV and RV in the intestine (Tarris et al., 2021). SNPs can determine the secretion status of *FUT2* gene, such as G428A (Caucasians population) (Soejima and Koda, 2021a), 778C>Del (Soejima and Koda, 2021a), A385T (Asian population) (Ye and Yu, 2021), 818C>A (Thr273Asn) (Soejima et al., 2012), 853G>A (Ala285Thr) (Soejima et al., 2012), 617 T>G (V206G) (Tian et al., 2014), 841G>A (G281R) (Tian et al., 2014), 244G>A (Soejima et al., 2008), 569G>A (Soejima et al., 2008), 950C>T (Soejima et al., 2008), 357C>T; 856T>C; and 863C>T (Soejima and Koda, 2021b); both are mutations that inactivate the enzyme and thus result in loss of Se enzyme activity, which mainly determines the non-secretor type. However, this is not a universal phenomenon in populations around the world, as SNPs of *FUT2* are specific for population and region. For instance, a study described that the homozygosity for a nonsense mutation G428A in the *FUT2* increases the risk of susceptibility to IBD in the Finnish population due to the absence of ABO blood groups in body fluids (Parmar et al., 2012). Similarly, a study from southeastern China suggested that *FUT2* gene polymorphisms were considered to be associated with susceptibility to Crohn's disease (CD) (Wu et al., 2017). In another study, mutant allele and genotype of *FUT2* (A385T and G428A) were dramatically increased in patients with CD (Wu et al., 2017). On the contrary, SNP 235G>A (Soejima and Koda, 2020), 304G>A (Soejima and Koda, 2020), 357C>T, 4G>A (Soejima et al., 2008), 489G>A (Soejima et al., 2008), and 665G>A (Soejima et al., 2008) were determined as the secretor type in a particular population, which were regarded as a protective factor for IBD, although it may be a risk locus for entering the virus genetically. As such, homozygous nonsense mutation (428G>A) in the *FUT2* gene provides resistance to symptomatic NoV (GGII) infections (Thorven et al., 2005). Indeed, as discussed, the secretor status of *FUT2* has shown a significant association with intestinal diseases.

## *FUT2* and IBD

### Crohn's Disease

Inflammatory bowel disease (IBD), including ulcerative colitis (UC) and CD, is a multifactorial disease in which dietary, genetic, immunological, and microbial factors are at play (Khor et al., 2011; Nishida et al., 2018).

Recently, numerous studies have been conducted suggesting the involvement of *FUT2* in genetic susceptibility to IBD, particularly CD. For instance, the association between *FUT2* polymorphism and susceptibility to CD was observed in a Belgian sample (Forni et al., 2014), and this study provides strong evidence that non-secretor status increases susceptibility to CD (McGovern et al., 2010). A study in a small Japanese cohort reported that the *FUT2* secretor status was also specifically associated with the colonic localization of CD (Miyoshi et al., 2011). Recently, an animal study using *FUT2*ΔIEC mice (*FUT2* gene knockout mice) found more serious intestinal inflammation and destructive barrier functions that occurred in *FUT2*ΔIEC mice compared with WT mice when DSS was treated; simultaneously, while at the same time, less diversity of intestinal microbiota was observed in *FUT2*ΔIEC mice (Tang et al., 2021). It is found that *N*-acetylgalactosamine, one of the compounds synthesized by HGBA, may change gut microbiota in pigs (Yang et al., 2022). Contributing to the up-to-date progress in next-generation sequencing technology, IBD patients have been found to have alterations in the composition and function of the gut microbiota, termed dysbiosis, which is thought to play a central role in the pathogenesis of IBD (McGovern et al., 2010). In addition, dysbiosis may be one of the reasons for the increased susceptibility (McGovern et al., 2010); in fact, the composition and function of the intestinal microbiota were suggested to be affected by HBGA synthesis, which is mediated by both the *FUT2* and *ABO* genes (Forni et al., 2014). The different secretor statuses result in the expression of ABO and Lewis glycan epitopes in the intestinal mucosa that may have affected the attachment of bacteria. For instance, probiotics *Bifidobacteria* (Wacklin et al., 2011) and *Lactobacillus* genus (Watanabe et al., 2010) show a significant decrease in the individuals with IBD genetic risk compared with healthy controls. Meanwhile, Rauschet al., indicated that compared with the secretor, the bacterial composition of non-secretors resembles more tightly to CD patients (Rausch et al., 2011). Given these observations, it comes as no surprise that the function of the *FUT2* gene could have a positive impact on dysbiosis of intestinal microbiota.

A defective immune response against the commensal intestinal microbiota has been proposed as one of the most possible pathogenic mechanisms of CD (Xavier and Podolsky, 2007).

### Ulcerative Colitis

According to the recent studies, the contribution of *FUT2* to the pathogenesis of UC remains, to date, less clear. McGovern et al., reported that the non-secretor status is associated only with CD, and the association between UC and *FUT2* is difficult to confirm (McGovern et al., 2010). Nevertheless, SNPs of the *FUT2* gene leading to non-secretor status, including rs281377, rs1047781, and rs601338, have been associated with UC in a small sample population of Han and Uyghur ethnic groups in China (Aheman et al., 2012). In contrast to what has been discussed above for CD, a report based on a Finnish population showed that the homozygous rs601338-AA (G428A) SNP indicates a dominant protective role of the A allele against UC, while the GG genotype showed an increased risk of UC. That is to say, the mutant allele appears to be more protective of UC than of CD (Parmar et al., 2012). There is no doubt that IBD is caused by both UC and CD, but the two diseases are quite different in their genetic susceptibility. For example, the secretor statuses of FUT2 lead to opposite outcomes in susceptibility of the two diseases and have complex interactions with the extra genetic, environmental, and other factors that cause disease severity. The same is true for CD and UC: genetic variation of FUT2 was closely related to changes in different gut microbiota in patients with IBD.

### The Hypothesis of Pathogenetic Mechanism of IBD: Interactions in Host-Microbe and Fucosylated Glycans

Although a few theories suggest that the taxonomic composition of the microbiota does not appear to be strongly associated with ABO or secretor status (Davenport et al., 2016), clinical and experimental data illustrate that dysbiosis could be a crucial element in the pathogenesis of IBD (Nishida et al., 2018). For instance, *Bifidobacteria* are important probiotics in the human intestine and the *Bifidobacteria*l population detectable in the intestine of a healthy adult always consists of one to four species under normal conditions (Mättö et al., 2004), while one study from the fecal samples of 71 healthy individuals with different secretor status found that the diversity and abundance of dominant bacteria and *Bifidobacteria* were considerably lower in the samples from the non-secretor individuals as compared with those from the secretor individuals (Wacklin et al., 2011); similarly, the *Lachnospiraceae* family with lower abundances was noted in nonsecretors (Gampa et al., 2017); in other words, the non-secretors were considered to have lower species richness than the secretors (Wacklin et al., 2014). The reason was potentially explained, as the different secretor statuses may result in the expression of ABO and Lewis glycan epitopes in the intestinal mucosa that may have affected the attachment of bacteria. Recently, in animal experiments, it has been found that N-acetylgalactosamine, which is one of the compounds synthesized by HGBA, may change gut microbiota in pigs (Yang et al., 2022). Similarly, the probiotic *Lactobacillus* genus may hinder the attachment of potential pathogens to mucosal surfaces by possessing adhesins that bind to the ABO blood group antigens in the mucosa (Watanabe et al., 2010). Another study of the genus *Roseburia* showed a significant decrease in the individuals with IBD genetic risk compared with healthy controls (Imhann et al., 2018).

From the abovementioned data, we can draw a hypothesis that the *FUT2* gene mediates the probiotic population by regulating the production of HBGA. Also, *Roseburia spp* functions to convert acetate into butyrate (Imhann et al., 2018), which protects against colon inflammation (Chang et al., 2014), counter-intuitively. Cheng et al., found more butyrate-producing bacteria and higher butyrate levels in *sese* individuals through metabolomic analysis (Cheng et al., 2021). This finding is against the abovementioned hypothesis. Thus, another hypothesis for the involvement of *FUT2* in the mechanism of IBD is that nonfunctional mutation in *FUT2* reduces the abundance of appressed bacteria by reducing their binding sites, which may allow the overgrowth of other bacteria, which in turn induces inflammatory T cells and leads to IBD (Cheng et al., 2021).

Given these observations, we agreed and emphasized the suggestion that the gut bacterial community in IBD patients and *FUT2* with non-secretors differs from one of the secretors or healthy individuals (Nishida et al., 2018). The dysfunction of *FUT2* is one of the most important causes of increased susceptibility to IBD including CD and UC. A brief summary of some variations and functions of common probiotic and pathogenic bacteria in IBD is shown in Table 1.

**Table 1:** Variation and function of common probiotics and pathogenic bacteria in IBD.

**Species**	**Changes in IBD patients**	**Function**
**Protective bacteria species**		
*Faecalibacterium prausnitzii*	amount↓	Anti-inflammatory and prevent colitis (Rossi et al., 2016)
*Roseburia intestinalis*	abundance↓	Acetate to butyrate converter (Vich Vila et al., 2018)
*Ruminococcaceae*	amount↓	Produce SBA (Lo Presti et al., 2019)
*Akkermansia muciniphila*	abundance↓	Partially (Png et al., 2010)
**IBD**		
*Phascolarctobacterium*	amount↓	Succinate utilizing bacterium (Watanabe et al., 2012)
*Odoribacter splanchnus*	amount↓	Producer of acetate, propionate and butyrate (Göker et al., 2011)
*Clostridium lavalense*	amount↓	Producing butyrate (Takahashi et al., 2016)
*VSL#3*	amount↓	Anti-inflammatory (Fedorak et al., 2015)
*E.coli Nissle 1917*	amount↓	Reinforcement of intestinal barrier (Scaldaferri et al., 2016)
*Blautia faecis*	amount↓	Produces butyrate (Takahashi et al., 2016)
**Causative bacteria species**		
*Roseburia hominis*	amount↑	Produce acylcarnitines (Lloyd-Price et al., 2019)
*Klebsiella pneumoniae*	amount↑	Produce acylcarnitines (Lloyd-Price et al., 2019)
*Haemophilus parainfluenzae*	amount↑	Produce acylcarnitines (Lloyd-Price et al., 2019)
*Clostridium bolteae*	amount↑	Produce acylcarnitines (Lloyd-Price et al., 2019)
*Escherichia coli*	amount↑	Diarrhea (Lloyd-Price et al., 2019)
*AIEC*	amount↑	Contribute to IBD pathogenesis (Sepehri et al., 2011)
*ETBF*	amount↑	Mucosal permeability↑ (Wick et al., 2014; Dejea et al., 2018)
*Campylobacter concisus*	amount↑	Enteropathogenic potential (Zhang et al., 2014)
*Fusobacterium varium*	amount↑	cause colonic mucosal inflammation (Sekizuka et al., 2017)
*Ruminococcus gnavus*	amount↑	Secrete pro-inflammatory complex polysaccharide (Henke et al., 2019)

*AIEC, adherent invasive Escherichia coli; ETBF, entero-toxigenicBacteroides fragilis*.

*↑, ↓ presented as the increase or decrease of amount and abundance of bacteria*.

## *FUT2* Gene and Susceptibility to NoV and RV Infections

### *FUT2* Gene and NoV Infections

Norovirus, an RNA virus of the Caliciviridae family, is known as a human enteric pathogen that causes substantial morbidity (Robilotti et al., 2015), and is the leading cause of nonbacterial gastroenteritis and acute gastroenteritis (AGE) worldwide (Haga et al., 2020). Unlike the susceptibility to IBD, HBGA expression under the influence of the *FUT2* gene is an important susceptibility risk for NoV infection, and secretor status is associated with susceptibility to NoV infection (Currier et al., 2015; Haga et al., 2020), while non-secretors lack enzyme coded by *FUT2* resulting in high resistance to infection and gastroenteritis caused by numerous NoV strains. Moreover, GII.4 NoV infects secretors exclusively (Currier et al., 2015); it is the most commonly detected genotype of NoV, and the same indication was found in a community-based birth cohort of 194 children in Ecuador (Lopman et al., 2015). The next major genotypes were GII.3 and GII.6 (Mans, 2019). However, the non-secreting form is resistant to several NoV genotypes. After all, the link between *FUT2* and NoV susceptibility has been explained by the fact that *FUT2* secretor status alters HBGA expression in the intestine, while some GII group genotypes, mainly GII.4 and GII.6, use H or Lewis antigens as the main binding saccharides mediated by fucosyl- and glycosyltransferases under the genetic control of *FUT2* (secretor), *FUT3* (Lewis), and *ABO(H)* genes (Chen et al., 2011). This is the reason why some NoV are more susceptible in individuals with secretor status genotype rather than the non-secretor one.

The second factor affecting enterovirus infection is the host gut microbiome. Similar to IBD, for example, probiotics protect the host from viral infection by modulating the composition of the gut microbiota, enhancing intestinal barrier function, and promoting mucosal immunity (Peña-Gil et al., 2021). Additionally, they interfere with the binding of viruses to target cells by blocking viral receptors and binding to the surface in a competitive rejection manner, facilitating their excretion in the feces (Monedero and Rodríguez-Díaz, 2016). Furthermore, the presence of *Bifidobacterium* adolescentis inhibits the attachment of NoV GI.1 VLPs to epithelial cells *in vitro* (Li et al., 2016). In another study, *Lactobacillus rhamnosus GG* (LGG) and *Escherichia coli Nissle 1917* (EcN) can bind NoV P particles containing genotype GII.3 and GII.4; this was investigated using the gnotobiotic pig model, and viral shedding in feces below the detection limit was observed, indicating significant inhibition on NoV infection by the colonization of such bacteria. Additionally, *Lactobacillus rhamnosus* GG (LGG) and *E. coli* Nissle 1917 (EcN) as a probiotic cocktail and supplemented with rice bran had a high protective effect against HuNoV diarrhea and shedding (Lei et al., 2016). There is also evidence which suggests that the members of the intestinal microbiota such as *Enterobacter cloacae, E. coli*, and *Helicobacter pylori* facilitate NoV infection (Rasko et al., 2000; Yi et al., 2005; Jones et al., 2014). However, studies on the negative effects of the microbiota on NoV infectivity are scarce. Epidemiological studies suggest that vitamin A intake has an anti-NoV impact. Research with murine NoV draws a conclusion that the amount of intestinal *Lactobacilly* ssp. increased after supplementation of vitamin A, which was speculated as the reason for the antiviral effect (Lee and Ko, 2016).

In brief, the abovementioned studies have clearly demonstrated that the major genotype GII.4, as well as several other less common genotypes, have clear specificity of secretor. A wide range of different NoV genotypes, including GI.3, GI.6, GII.1, GII.2, and GII.7 (Nordgren et al., 2013; Prystajecky et al., 2014; Currier et al., 2015; Lopman et al., 2015), have also been described in these studies that are also capable to infect an individual with non-secretors genotypes. However, no genotype has been found to exclusively infect non-secretors. In the previous discussion, the alteration of intestinal flora has been considered to be an important cause or consequence of intestinal diseases, and there are hypotheses suggesting that its function and attachment can be differentially influenced by FUT2, which has received great attention in the previous studies on IBD, and at the same time, some genera are also associated with viral infections, so it should also receive more attention in the studies about NoV, especially the pathogenic mechanism.

### *FUT2* Gene and RV Infections

Rotavirus (RV) is the most common causative agent of severe dehydrating gastroenteritis in children worldwide (Sharma et al., 2020). To some extent, the susceptibility effect of the *FUT2* gene of RV is similar to that of NoV. HBGAs act as important attachment factors for human RV, especially VP8^*^ (P-genotype) (Günaydin et al., 2016; Sharma et al., 2020). VP8^*^ is the glycan-binding domain of VP4 protein (P genotype), which is used to define protease-sensitive (P) serotypes or genotypes; structural proteins (VP) were encoded by double-stranded RNA gene segments in triple-layered capsid (Sharma et al., 2020). In fact, the function of VP8^*^ is responsible for binding to cellular receptors, namely HBGA, thereby facilitating viral attachment and entry (Sharma et al., 2020). Moreover, a French study suggested that RV P[8] infections were completely absent in individuals with the non-secretor phenotype, namely, the homozygous nonsense mutation *FUT2* (Imbert-Marcille et al., 2014). In addition, a study based on children in Burkina Faso and Nicaragua found that P[8] and P[4] genotypes barely infected non-secretor and Lewis-negative children, while P[6] RV mostly infected children whose phenotype was independent of secretor status yielding the Lewis-negative population (Nordgren et al., 2014). However, most studies have suggested a strong association between positive secretor status and susceptibility of RV; some researchers have found individuals with Lewis positivity, independent of secretor status phenotype, as a susceptibility factor (Ayouni et al., 2015), while others have found that the individuals have both secretor- and Lewis-positive statuses, as well as Lewis b phenotype seemingly has a higher susceptibility risk than only secretor-positive status (Nordgren et al., 2014).

In terms of bacteria, RV-induced diarrhea may be reduced *via* an immune response, modulated by *E. coli Nissle 1917* (Kandasamy et al., 2016; Vlasova et al., 2016). In addition, *Ruminococcus gauvreauii*, namely, a bacterium that has been isolated from human bile and seems to present in the small intestine, which is the site more likely to infect RV, has also been proved that can bind RV. Viral infectivity was reduced to threefold in the presence of *R. gauvreauii*, indicating that this bacterium has an anti-RV effect (Gozalbo-Rovira et al., 2021). Moreover, bacteria belonging to *lactobacilli, Mucispirillum, Oscillospira*, and *Bilophila* genera were negatively linked to RV infection in mice (Peña-Gil et al., 2021). In fact, the effect of HGBA on bacterial attachment, which may lead to interference with RV infection *in vitro*.

To summarize, we can conclude that secretor and Lewis antigens are important for susceptibility to RV such as in a P genotype–dependent way.

## Discussion

As mentioned above, growing evidence and hypotheses support the involvement of *FUT2* in the pathogenesis of many intestinal diseases. Among them, various inactivating polymorphisms resulting in a non-secretor phenotype is a susceptibility factor for IBD and conversely a protective factor for NoV and RV, the reasons behind which are due to the presence or absence of HBGA, in which synthesis is regulated by the *FUT2* gene. The hypothesis explains that the *FUT2* gene may regulate the synthesis of HBGA at the attachment site of intestinal bacteria, thus enabling the screening of the flora. An important factor in intestinal health is the flora that has been hypothesized to be influenced by the sugar chain called HBGA. Therefore, there is a non-negligible association between the *FUT2* gene and intestinal diseases including IBD, which is caused by RV and NoV.

The disease caused by SARS-CoV-2 was subsequently named as COVID-19 by the WHO. Since the rapid global outbreak of COVID-19, extensive research suggested the ABO blood group system and secretor phenotype (Valenti et al., 2020) in potentially predisposing to infection with SARS-CoV-2 (Al-Youha et al., 2021). To date, a meta-analysis illustrates that the association between the ABO blood group and the infection of COVID-19 is that blood type A might be more susceptible to infecting COVID-19, while blood type O might be less susceptible to infecting COVID-19 (Wu et al., 2020). But the effect of the ABO blood group on the COVID-19 is not only in the infection but also contains severity and demise (Torres-Alarcón et al., 2021). A Chinese study analysis association between the ABO blood group and COVID-19 suggested that there was a statistically significant difference for blood type A rather than blood types B, AB, or O (Liu et al., 2021), particularly in women of Wuhan (Fan et al., 2020). The changes in host susceptibility to many infections are actually because of diversities in blood group antigen expression (Cooling, 2015). That is why more and more researches found out that the O blood type is a protection factor against COVID-19 infection and severe COVID-19 illness (Flegel, 2020; Wu et al., 2020; Liu et al., 2021; Ray et al., 2021), whereas a study reported that the interactions of the ABO and secretor types in the cohort of patients compared with the blood donors lack significant differences, so they suggested that that emphasis should not be put on the ABO antigenic structures, but rather on the isoagglutinin (Matzhold et al., 2021). In addition to using different antigens, different ABO blood groups also contain different situations of isoagglutininsin. ABO blood group O, with obligatory anti-A and anti-B antibodies present, is protective against COVID-19 (Matzhold et al., 2021). The Le (a–b–) type appeared to be at least a mitigating factor against hospitalization with COVID-19. Invariably, many study results reach a consensus that the O blood type acts as a protection factor against COVID-19, while no ABO blood group type can protect an individual from becoming infected (Flegel, 2020). Notably, individuals able to express these antigens on epithelial cells, known as the *FUT2* gene secretion status, indicated a higher degree of exposure to SARS-CoV-2 (Shokri et al., 2022).

Moreover, mounting evidence currently suggested that neurological diseases including Parkinson's disease (PD) (Brudek, 2019; Lee et al., 2021) anxiety, and depressive disorder (Barberio et al., 2021) were highly related to the IBD. In addition, the gut microbiome is considered to be an indispensable element in the interaction between IBD and neurological disease (Needham et al., 2020; Nielsen et al., 2021). Currently, the existence of the brain-gut axis has been described by an increasing number of studies and is one of the most accepted reasons for this phenomenon (Sun and Shen, 2018; Carloni et al., 2021). The metabolites of the flora may enter certain areas of the brain through the brain-gut axis and affect the regular function of a specific brain. In the study of PD, the gut microbiome is required for microglial activation, α synuclein pathology, and motor deficits, which is a focus of research on the pathogenesis of PD (Nielsen et al., 2021). Whereas a possible chemical metabolite, 4-ethyl phenyl sulfate (4EPS), produced by intestinal bacteria from tyrosine metabolism (Hsiao et al., 2013), was identified in studies of depression and anxiety; 4EPS enters the mouse brain and affects the activation and connectivity of specific brain regions, disrupting brain oligodendrocyte maturation and myelination patterns, thereby modulating brain activity and anxiety-like behavior in mice. As a result, mice exposed to 4EPS exhibited anxiety-like behaviors and pharmacological treatment promoting oligodendrocyte differentiation blocked by the behavioral effects of 4EPS; pharmacological treatments that promote oligodendrocyte differentiation prevented the behavioral effects of 4EPS (Needham et al., 2022). In the future, animal studies using oral microbiota transplants from human PD cases could provide further insight into the entire mechanism. Therefore, the *FUT2* gene, as mentioned above, has the function of regulating intestinal flora that may also affect the diseases associated with IBD, which can be regarded as a new research direction.

## Author Contributions

MH, XZ, JL, and TS initiated the project, wrote, revised, and finalized the manuscript. LC, XH, and TS searched the database. All authors contributed to the article and approved the submitted version.

## Funding

This study was financially supported by the National Natural Science Foundation of China (grant no. 32101226), the Young Elite Scientist Sponsorship Program by CAST (No. YESS20210189), the National Natural Science Foundation of China Program for Changjiang Scholars and Innovative Research Team in University (No. IRT_16R32), and the Science Foundation of Education Bureau of Jilin Province (Changchun, China; grant no. JJKH20211055KJ).

## Conflict of Interest

The authors declare that the research was conducted in the absence of any commercial or financial relationships that could be construed as a potential conflict of interest.

## Publisher's Note

All claims expressed in this article are solely those of the authors and do not necessarily represent those of their affiliated organizations, or those of the publisher, the editors and the reviewers. Any product that may be evaluated in this article, or claim that may be made by its manufacturer, is not guaranteed or endorsed by the publisher.
